# Welcome to *Skeletal Muscle*

**DOI:** 10.1186/2044-5040-1-1

**Published:** 2011-01-24

**Authors:** David J Glass, Kevin P Campbell, Michael A Rudnicki

**Affiliations:** 1 Novartis Institutes for Biomedical Research, Cambridge, MA, USA; 2 Howard Hughes Medical Institute, Carver College of Medicine, University of Iowa, Iowa City, IA, USA; 3 Ottawa Hospital Research Institute and University of Ottawa, Ottawa, ON, Canada

## 

Over the past decade or two, cellular signaling and molecular genetics have combined to allow for a tremendous increase in the understanding of fundamental processes that are distinct to skeletal muscle: the genes that initiate its differentiation from progenitor cells and which establish its identity; pathways that are responsible for its hypertrophy under load and its atrophy in settings of cachexia, disuse and denervation; an increase in the understanding of its remarkable and distinct ability to regenerate from cellular injury and how that ability declines with age; the genes that are responsible for the multiple types of muscular dystrophy and some indication as to how these genes function; a finer understanding of muscle contractility and the signaling pathways that control contraction; the role of the mitochondria and energy use in skeletal muscle; the contribution of skeletal muscle to insulin and fatty acid signaling; and the discovery of precursor cells that can give rise to skeletal muscle, most notably the satellite cell. Beyond these basic and exciting discoveries, there are also the complex interactions between muscle and motor neuron, muscle and fat, muscle and vasculature, muscle and tendon, and muscle and bone, that are becoming subjects of study. In addition, researchers have begun to explore how skeletal muscle's basic processes are perturbed in settings of disease and advanced age. The dramatic increase in research on skeletal muscle richly justifies the establishment of a new home for the resultant reports detailing progress in the understanding of this remarkable tissue.

*Skeletal Muscle *is therefore launched to provide such a home. We should emphasize that we take that metaphor seriously. The pages of this journal will provide space in the same way a home in a community functions: to give the active scientist room to develop a field; to report exciting new understandings as to mechanisms that constitute and control skeletal muscle; to distribute notes on important techniques; to review recent developments; to provide perspective on long-standing research; and to provide constructive feedback from peers well versed in the research area. The journal should be the first resort for an experienced scientist researching skeletal muscle who seeks to reach other scientists who would be most interested in a finding. It is hoped that this journal will become required reading for students who want to learn about skeletal muscle and for experienced scientists who seek to keep up with the most important developments in the field. We have a goal that this journal will become the skeletal muscle equivalent of *Neuron *or *Blood*, to name other journals that provide similar space for research in their named tissue types. The reason *Skeletal Muscle *is needed is simply that no similar journal for skeletal muscle exists.

Ours is an open access journal [1]. Open access will allow key findings in our field to be more accessible and far-reaching, because the journal will be published online and without subscription charges. An online journal means more rapid reviews and shorter time to publication. For a growing field, dispensing new knowledge quickly is critical. The online format will also allow us to publish articles without the constraint of page limitations. A fixed article- processing charge will be levied to cover the publication costs [2], and there will be no additional page or color figure charges. Furthermore, the active nature of the contracting or regenerating skeletal muscle system is often well captured in the form of movies and animation. We have made this a priority in the format of *Skeletal Muscle*: movies may be built into an article, such that readers will be only a click away from a real-time view of the observations. Also, the online format permits the journal to generate a cover for every article we publish (and contributors are invited to furnish cover art for their papers).

The editors are committed to making this new venture a success for the skeletal muscle community [3]. We therefore hope that you share our vision and will join us in launching *Skeletal Muscle*. We will not be successful without your enthusiastic support and contributions.
